# Chemogenetic activation of G_12_ signaling enhances adipose tissue browning

**DOI:** 10.1038/s41392-023-01524-2

**Published:** 2023-08-21

**Authors:** Yuki Ono, Ryo Ito, Kaito Arai, Gurdeep Singh, Tsuyoshi Saitoh, Robert B. Russell, Francesco Raimondi, Junken Aoki, Juro Sakai, Asuka Inoue

**Affiliations:** 1 Molecular and Cellular Biochemistry, Graduate School of Pharmaceutical Sciences, Tohoku University, Sendai, Miyagi 980-8578 Japan; 2 Division of Molecular Physiology and Metabolism, Graduate School of Medicine, Tohoku University, Sendai, Miyagi 980-8574 Japan; 3 Bioquant, Heidelberg University, Im Neuenheimer Feld 267, 69120 Heidelberg, Germany; 4 Biochemie Zentrum Heidelberg (BZH), Heidelberg University, Im Neuenheimer Feld 328, 69120 Heidelberg, Germany; 5 International Institute for Integrative Sleep Medicine (WPI-IIIS), University of Tsukuba, 1-1-1 Tennodai, Tsukuba, Ibaraki 305-8575 Japan; 6 Graduate School of Comprehensive Human Sciences, University of Tsukuba, 1-1-1 Tennodai, Tsukuba, Ibaraki 305-8575 Japan; 7 Laboratorio di Biologia Bio@SNS, Scuola Normale Superiore, Piazza dei Cavalieri 7, Pisa, 56126 Italy; 8 Department of Health Chemistry, Graduate School of Pharmaceutical Sciences, The University of Tokyo, Bunkyo-ku, Tokyo 113-0033 Japan; 9 Division of Metabolic Medicine, Research Center for Advanced Science and Technology, The University of Tokyo, Meguro-ku, Tokyo 153-8904 Japan

**Keywords:** Drug discovery, Biological techniques, Chemical biology

**Dear Editor**,

Beige adipocytes, which increase energy expenditure by dissipating energy as heat, have gained attention as a therapeutic target for combating obesity.^1^ Adipocytes express many types of G-protein-coupled receptors (GPCRs), each of which has a unique preference for the G_s_, G_i_, G_q_, and G_12_ subfamilies. While the function of G_s_-coupled β-adrenergic receptors in beige adipocyte induction is well established,^2^ little is known about the function of G_12_-coupled GPCRs beyond its suppressive roles in white adipocyte maturation.^3^ In this study, we generated transgenic mice conditionally expressing a G_12_-coupled designer GPCR using a Cre-loxP system and investigated the potential effects of G_12_ signaling on adipocyte biology.

We first sought to improve the G_12_-coupling selectivity of the previously established G_12_-coupled Designed Receptor Exclusively Activated by Designer Drugs (DREADD; M3D-GPR183/ICL3), which showed a leaky coupling to other G-protein subtypes such as G_o_.^4^ Using PRECOG,^5^ a GPCR-coupling prediction algorithm, we designed six single-point mutants and assessed G-protein-coupling activity upon stimulation with the designer ligand, clozapine N-oxide (CNO) (Supplementary Fig. 1a). The F^1.57^V mutant (superscript denotes Ballesteros-Weinstein numbering) reduced off-target G_o_ coupling while maintaining G_12_ coupling and a surface expression level (Fig. 1a, Supplementary Fig. 1b). G-protein-coupling profiling of the F^1.57^V construct revealed preferential activation of G_12_ among the four G-protein subfamilies (Fig. 1b). We chose the F^1.57^V mutant of the M3D-GPR183/ICL3 construct (hereafter referred to as G_12_D) for the following transgenic mouse study.

**Fig. 1:**
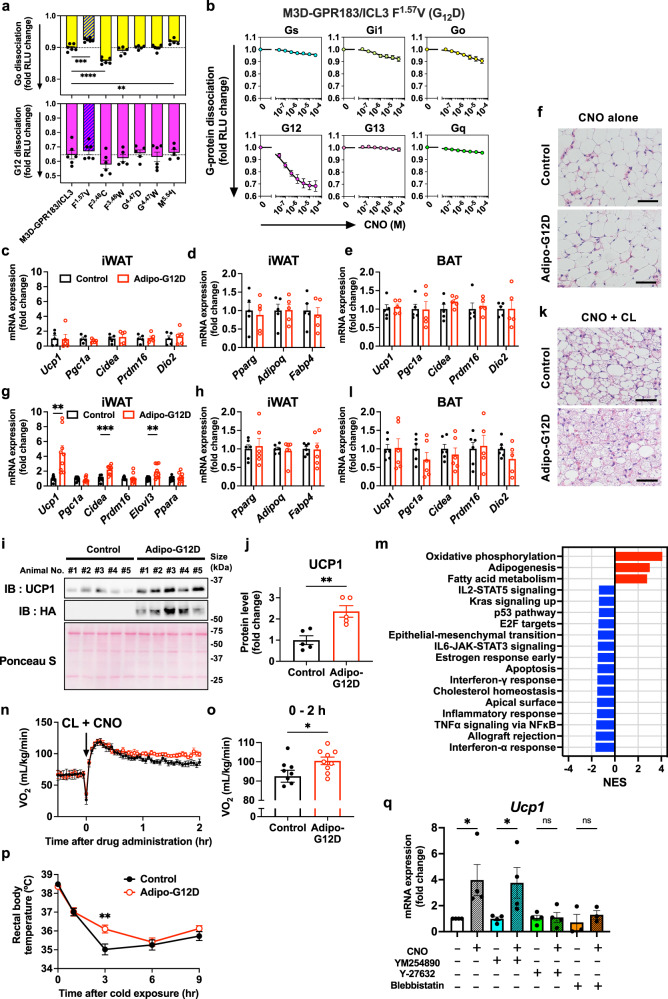
Synergistic induction of beige adipogenesis through the G_12_-coupled designer GPCR and the G_s_-coupled β3AR. **a** Functional screening of M3D-GPR183/ICL3-derived constructs for G_12_ selectivity. G_12_ and G_o_ activation was measured by the NanoBiT-G-protein dissociation assay with 10 µM CNO. Note that G-protein activation causes decrease in luminescence counts. Bars and error bars represent the mean and SEM, respectively, of 5-6 independent experiments (dots) with each performed in duplicate. The F^1.57^V mutant of the M3D-GPR183/ICL3 construct (oblique bars) is referred to as G_12_-DREADD or G_12_D. **b** Concentration-response curves for G-protein activation by the F^1.57^V M3D-GPR183/ICL3 construct. The G-protein-coupling profile was examined by the NanoBiT-G-protein dissociation assay using representative members of the four G-protein subfamilies. Symbols and error bars represent the mean and SEM, respectively, of 3–9 independent experiments with each performed in duplicate. **c**, **d** Expression of thermogenic (**c**) and adipogenic (**d**) genes in iWAT following CNO single-drug administration (1 mg/kg, i.p., daily) for 5 days (*n* = 5 per group). **e** Expression of thermogenic genes in BAT following CNO administration (1 mg/kg, i.p., daily) for 5 days (*n* = 5 per group). **f** Representative H&E staining of iWAT following CNO single-drug administration (1 mg/kg, i.p., daily) for 5 days (scale bar: 50 µm). **g**, **h** Expression of thermogenic (**g**) and adipogenic (**h**) genes in iWAT following CL316,243 and CNO dual-drug administration (1 mg/kg each, i.p., daily) for 5 days (*n* = 9 for (**g**) and 6 for (**h**) per group). **i**, **j** Ucp1 protein levels detected by immunoblotting in iWAT of the control and the adipo-G_12_D mice treated dually with CL316,243 and CNO (1 mg/kg each, i.p., daily) for 5 days (*n* = 5 per group). **k** Representative H&E staining of iWAT following CL316,243 and CNO dual-drug administration (scale bar: 50 µm). **l** Expression of thermogenic genes in BAT following 1 mg/kg CL316,243 and 1 mg/kg CNO dual-drug administration for 5 days (*n* = 5 or 6 per group). **m** Significantly enriched gene sets (FDR < 0.05) in GSEA. Red: positive NES, blue: negative NES. **n**, **o** Whole-body energy expenditure. The mice that were pretreated dually with CL316,243 and CNO (1 mg/kg each, i.p., daily) for 5 days were placed in the metabolic chamber and oxygen consumption rate (VO_2_) was monitored before and after the dual-drug administration (CL316,243 and CNO, 1 mg/kg each, i.p.; *n* = 9). Average VO_2_ during post 2-h drug administration (**o**). **p** Rectal body temperature of the mice treated dually with CL316,243 and CNO (1 mg/kg each, i.p., daily) for 5 days upon acute exposure to 4 °C for the indicated time (*n* = 12 per group. **q** *Ucp1* expression in primary SVF cells stimulated with 10 µM CNO, with or without pretreatment of 10 µM YM-254890, 10 µM Y-27632, or 50 µM blebbistatin (*n* = 3 or 4 per group). In all figure panels, bars or symbols, and error bars represent mean and SEM, respectively. Statistical significance was determined by one-way ANOVA followed by the Dunnett’s post-hoc test (**a**), the two-tailed Student’s *t*-test (**c**–**e**, **g**, **h**, **j**, **l**), the two-way ANOVA followed by the Sidak’s post-hoc test (**p**) or the one-way ANOVA followed by the Sidak’s post-hoc test (**q**). *P < 0.05, **P < 0.01, ***P < 0.001, and ****P < 0.0001

We generated mice expressing HA epitope-tagged G_12_D in adipocytes (adipo-G_12_D mice) by crossing the *Rosa26-LSL-G12D-IRES-GFP* mice (Supplementary Fig. 2a) with the *Adipoq-Cre* mice. Western blot analysis confirmed its selective expression in adipose tissue (Supplementary Fig. 2b). In the basal state (without CNO administration), body weights and adipose tissue weights were not significantly different between the adipo-G_12_D mice and their control littermates (the *Rosa26-LSL-G12D-IRES-GFP* mice) (Supplementary Fig. 2c–e).

We next tested the effects of chemogenetic G_12_ activation on white-adipose tissue (WAT) browning and glucose homeostasis. Both control and adipo-G_12_D mice were treated with daily intraperitoneal (i.p.) injections of CNO (1 mg/kg) for 5 days prior to tissue collection. RT-PCR analysis showed that the expressions of thermogenic and adipogenic genes in inguinal WAT (iWAT) (Fig. 1c, d) as well as thermogenic genes in brown-adipose tissue (BAT) (Fig. 1e) were not significantly different between the two genotypes. Consistent with these observations, hematoxylin-eosin (H&E) staining showed no obvious morphological changes in both iWAT and BAT (Fig. 1f, Supplementary Fig. 3a). Furthermore, glucose tolerance was unchanged between the two genotypes in both regular chow- and high-fat diet (HFD)-fed conditions (Supplementary Fig. 4a, b). Therefore, activation of G_12_ signaling alone does not affect WAT browning, BAT activation, or whole-body glucose homeostasis.

We then investigated the synergistic effects of G_12_ activation with G_s_ activation through β3AR stimulation. Chronic administration of the β3AR-selective agonist CL316,243 is a widely used method to induce WAT browning and BAT activation in mice. Both groups of the mice were administered with CL316,243 together with CNO (both 1 mg/kg i.p.) daily for 5 days and evaluated for WAT browning. qRT-PCR analysis revealed a significant upregulation of key thermogenic genes (*Ucp1, Cidea*, and *Elovl3*), but not adipogenic genes, in iWAT of the adipo-G_12_D mice (Fig. 1g, h). In the absence of CNO, the induction of the thermogenic genes was comparable between the genotypes (Supplementary Fig. 3f). Western blotting validated increased UCP1 protein levels in the adipo-G_12_D mice (Fig. 1i, j). H&E staining demonstrated the increased numbers of multilocular cells, typical morphology of beige adipocytes in the adipo-G_12_D mice (Fig. 1k, Supplementary Fig. 3d, e). In contrast to the effects observed in iWAT, BAT showed minimal synergistic effects of CNO and CL316,243 on thermogenic gene expression and histology (Fig. 1l, Supplementary Fig. 3b, c). To further analyze the pathways affected by G_12_D activation, we performed RNA-seq transcriptome analysis using iWAT RNA samples. Gene set enrichment analysis (GSEA) revealed that genes upregulated in the adipo-G_12_D mice were involved in oxidative phosphorylation, adipogenesis, and fatty acid metabolism pathways (Fig. 1m, Supplementary Fig. 5), which play key roles in the development of beige adipocytes. The emergence of the adipogenesis characteristics in the analysis is likely attributable to upregulation of genes involved in oxidative phosphorylation and fatty acid metabolism, because these genes also fall under the “adipogenesis” category in the GSEA. Downregulated gene sets were associated with the inflammatory response, such as interferon-α response and TNF-α signaling via NF-κB (Fig. 1m, Supplementary Fig. 5), which is involved in adipocyte dysfunction, including insulin resistance.

To explore the physiological significance of G_12_D-enhanced WAT browning, we evaluated whole-body energy expenditure and adaptive thermogenesis. After simultaneous daily injections of CNO and CL316,243 (both 1 mg/kg i.p.) for 5 days at room temperature, the mice were placed in a metabolic chamber and oxygen consumption was monitored before and after the additional CNO and CL316,243 administration. An acute increase in oxygen consumption was observed in both groups of the mice following the drug administration, a phenomenon attributable to β3AR stimulation (Fig. 1n). In the adipo-G_12_D mice, the elevated oxygen consumption persisted over time, contrasting with the gradual decrease observed in the control mice (Fig. 1n, o). Under this condition, the blood glucose level was higher in the adipo-G_12_D mice while the free-fatty acid level was lower and the glycerol level was unchanged (Supplementary Fig. 6a–d), suggesting that β3AR-induced lipolysis remains unchanged, but fatty-acid uptake is enhanced in the adipo-G_12_D mice. To examine adaptive thermogenesis, after the 5-day drug administration, the mice were placed in a 4 °C cold chamber and and their rectal body temperature was measured. While the control mice showed a rapid decrease in body temperature (peak at 3 h), the decrease rate in the adipo-G_12_D mice was slower (peak at 6 h) (Fig. 1p). Together, these results demonstrate functional significance of G_12_D-induced beige adipocytes.

To investigate whether G_12_D-induced enhancement of WAT browning is controlled in a cell-autonomous manner and to understand the downstream mechanism, we performed a primary culture experiment. Stromal vascular fraction (SVF) was isolated from the adipo-G_12_D mice and differentiated into beige adipocytes in vitro. After differentiation, beige adipocytes were stimulated with CNO in the presence or absence of a series of signaling inhibitors (a Rho kinase (ROCK) inhibitor Y-27632, a G_q_ inhibitor YM-254890, or a myosin II inhibitor blebbistatin). Stimulation by CNO enhanced *Ucp1* expression (Fig. 1q), indicating that G_12_D-mediated *Ucp1* expression was at least partially cell-autonomous and that constitutive G_s_ signaling was induced in the culture condition. Furthermore, the enhancement of *Ucp1* expression was completely inhibited by pretreatment with Y-27632 and blebbistatin, but not by YM-254890 (Fig. 1q). This result demonstrates that G_12_D-mediated *Ucp1* expression is dependent on ROCK and myosin II, canonical downstream effectors of G_12_, and is not mediated by potential coupling of G_12_D to G_q_.

Since activation of G_12_ signaling by the DREADD system promoted WAT browning, we searched for G_12_-coupled GPCRs that are endogenously expressed in adipocytes. Using previously published RNA-seq data of isolated mouse iWAT adipocytes, we identified 16 types of G_12_-coupled GPCRs that are expressed in adipocytes (Supplementary Fig. 7).

In conclusion, we used our newly generated the adipo-G_12_D mice to elucidate the effect of chemogenetic activation of G_12_ signaling in adipocytes. The G_12_ signaling was found to synergistically enhance the beige adipogenesis triggered by the G_s_-coupled β3AR, thus potentiating the thermogenic effect in vivo. Although we hypothesize that the underlying mechanism is attributed to the synergistic effect downstream of G_s_ and G_12_ signaling, it is also conceivable that G_12_ signaling boosts the availability of cell-surface β3AR. Moreover, a study using the adpo-G_12_D mice in the G_12_ (*Gna12*)-deficient background will serve as an important validation, which we plan to investigate in the future. Nevertheless, our finding highlights a previously unrecognized role for G_12_ signaling as a regulatory pathway of beige adipocyte induction. As G_12_ signaling remains uncharacterized in many other tissues, the use of the Cre-driven G_12_D mice will expedite understanding of G_12_ signaling in physiology and pharmacology as well as drug development for G_12_-coupled GPCRs.

### Supplementary information

Supplementary materials

## Data Availability

All data generated in this study are included in the Source Data file. The RNA-seq data are viewable under the DDBJ accession number PRJDB14356.
