# Supplementary material for: Chemogenetic activation of G12 signaling enhances adipose tissue browning
Source: Signal Transduct Target Ther. 2023 Aug 21;8:307. doi: 10.1038/s41392-023-01524-2 (PMC10440338; doi:10.1038/s41392-023-01524-2)
Supplement: Supplementary file 1 — Supplementary materials [file 41392_2023_1524_MOESM1_ESM.pdf]

Supplementary Materials for

Chemogenetic activation of G_{12} signaling enhances adipose tissue browning.

Yuki Ono, Ryo Ito, Kaito Arai, Gurdeep Singh, Tsuyoshi Saitoh, Robert B. Russell, Francesco Raimondi, Junken Aoki, Juro Sakai, Asuka Inoue

Correspondence to: iaska@tohoku.ac.jp

This PDF file includes:

Materials and Methods
Figures S1 to S7
Table S1

Materials and methods

Materials

CNO dihydrochloride was synthesized at the International Institute for Integrative Sleep Medicine (WPI-IIIS), University of Tsukuba. CL316,243 dihydrochloride (Cat. 1499) were purchased from Tocris. YM-254890 and (-)-blebbistatin were purchased from Fujifilm Wako Pure Chemical. Y-27632 dihydrochloride was purchased from Enzo Life Sciences (ALX-270-333). pR26 CAG/GFP Asc was a gift from Ralf Kuehn (Addgene plasmid # 74285; http://n2t.net/addgene:74285 ; RRID:Addgene_74285)[1]. Plasmids used in NanoBiT-G-protein-dissociation assay were previously described[2]. For the HEK293 cell experiments, M3D-GPR183/ICL3-derived mutants were N-terminally FLAG-epitope-tagged and inserted into the pcDNA3.1 expression vector[2]. For the transgenic mice, $G_{12}D$ (M3D-GPR183/ICL3 $F^{1.57}V$) was N-terminally HA-epitope-tagged. The complete amino-acid sequence of $G_{12}D$ is following (the HA epitope tag is underlined; the altered sequences from the human M3 receptor are double-underlined, which includes $Y^{3.33}C$ and $A^{5.46}G$ in the ligand-binding pocket to engineer the receptor responsive to CNO, but unresponsive to the endogenous ligand acetylcholine):

MYPYDVPDYATLHNNSTTSPLFPNISSSWIHSPSDAGLPPGTVTHFGSYNVSRAAGNFSSPDGTT
DDPLGGHTVWQVVFIAFLTGILALVTIIGNILVIVSVKVNKQLKTVNNYFLLSLACADLIIGVISM
NLFTTYIIMNRWALGNLACDLWLAIDCVASNASVMNLLVISFDRYFSITRPLTYRAKRTTKRAG
VMIGLAWVISFVLWAPAILFWQYFVGKRTVPPGECFIQFLSEPTITFGTAIAGFYMPVTIMTILYW
RIYKETERTAKQNPLTEKSGVEKKAAQTLSAILLAFIITWTPYNIMVLVNTFCDSCIPKTFWNLG
YWLCYINSTVNPVCYALCNKTFRTTFKMLLLCQCDKKKRRKQQYQQRQSVIFHKRAPEQAL

Synthesis of 4-(8-chloro-5H-dibenzo[b,e][1,4]diazepin-11-yl)-1-methylpiperazine 1-oxide1 (CNO)

CNO was synthesized as described previously[3]. All commercially available chemicals and solvents were used without further purification. In general, reaction mixtures were magnetically stirred at the respective temperature under argon atmosphere. Reactions were monitored by thin layer chromatography (TLC). TLC and preparative TLC were carried out on silica gel plates [Kieselgel 60 F254 (0.25 mm and 0.50 mm), E. Merck AG, Germany], in which existing compounds were visualized by UV light (254 nm) and staining with phosphomolybdic acid in a sulfuric acid aqueous solution and ninhydrin in an ethanol solution followed by heating. Flush column chromatography was performed on silica gel (Fuji Silysia Chemical Ltd., 40–50 μm, spherical, neutral, CHROMATOREX PSQ60B). ^{1}H and ^{13}C NMR spectra were recorded on a JEOL JNM-ECS 400 instruments (1H: 400 MHz; 13C: 100 MHz). Chemical shifts are quoted in ppm using tetramethylsilane (δ = 0.00 ppm), CD_3OD (δ = 49.0 ppm) for 13C NMR spectroscopy. Signal patterns are indicated as br = broad; s = singlet; d = doublet; t = triplet, quin = quintet, m = multiplet.

Infrared spectra (IR) were recorded with a JASCO FT/IR-4100 spectrophotometer. Mass spectra were measured with a JEOL JMS-T100LP spectrometer. A melting point were determined on a Yanaco MP-500P melting point (Mp) apparatus and were uncorrected. Chemical names were generated using ChemDraw 21.0.

To a solution of Clozapine (5.00 g, 15.3 mmol) in MeOH (120 mL) was added slowly a solution of m-chloroperoxybenzoic acid (4.06 g, 15.3mmol) in MeOH (30 mL) at 0 ˚C under an air. After being stirred for 15 min at room temperature, triethylamine (4.24 mL, 30.4 mmol) was added slowly at room temperature. The reaction mixture was concentrated under reduced pressure. The crude residue was purified by column chromatography on silica gel ($CHCl_3$/MeOH = 91:9 to 80:20) to afford CNO (5.16 g, 98%) as a yellow solid.

IR (KBr): 3422, 1609, 1562, 1460, 1378, 972 cm^{-1}; ^1H NMR (400 MHz, CD_3OD): δ (ppm) 3.12-3.19 (m, 2H), 3.23 (s, 3H), 3.53-3.65 (m, 2H), 3.68-3.79 (m, 2H), 3.80-3.96 (m, 2H), 6.81 (d, J = 8.2 Hz, 1H), 6.87 (dd, J = 8.2, 2.3 Hz, 1H), 6.97 (d, J = 2.3 Hz, 1H), 7.01 (dd, J = 7.8, 0.9 Hz, 1H), 7.05 (dt, J = 7.8, 7.3, 0.9 Hz, 1H), 7.31 (dd, J = 7.8, 1.4 Hz, 1H), 7.36 (dt, J = 7.8, 7.3, 1.4 Hz, 1H). One proton (NH) was not observed; ^{13}C NMR (100 MHz, CD_3OD): δ (ppm) 43.2 (br, 2C), 60.2, 65.8 (2C), 121.4, 121.5, 123.9, 124.2, 124.9, 127.3, 129.6, 131.1, 133.7, 142.7, 143.2, 155.5, 164.1; HRMS–ESI (m/z): $[M+Na]^+$ calcd for $C_{18}H_{19}ClN_4NaO$: 365.1145, found: 365.1150.

Hydrochloride salt of CNO was obtained by the following method: To a stirred solution of CNO in $CHCl_3$/MeOH = 80:20 (50 mL) was added hydrogen chloride (1 M in Et_2O, 80 mL), and the reaction mixture was stirred at 0°C for 30 min. The resulting white precipitate was collected by filtration to give CNO・2HCl.

mp (dec.) 247–250 °C.; *Anal.* Calcd for $C_{18}H_{19}N_4OCl{\cdot}2HCl{\cdot}3.3H_2O$: C, 45.50; H, 5.85; N, 11.79. found C, 45.38; H, 5.61; N, 11.70.

NanoBiT-G-protein-dissociation assay

NanoBiT-G-protein-dissociation assay was performed as previously described[18]. For the screening experiment, plasmid transfection was performed in a 6-well plate with a mixture of 100 ng Gα-Lg-encoding plasmid, 500 ng $G\beta_1$-encoding plasmid, 500 ng Sm-$G\gamma_{t1}$-encoding plasmid, and 200 ng GPCR-encoding plasmid with (for G_{12} assay) or without (G_o assay) 100 ng of RIC8A-encoding plasmid (per well). For the G-protein-profiling experiment, plasmid transfection was performed using following combinations: Gαs-Lg, Sm-$G\beta_1$, $G\gamma_2$ and RIC8B (G_s); $G\alpha_{i1}$-Lg, Sm-$G\beta_5$ and $G\gamma_2$ (G_{i1}); $G\alpha_o$-Lg, Sm-$G\beta_1$ and $G\gamma_2$ (G_o); $G\alpha_q$-Lg, Sm-$G\beta_1$, $G\gamma_2$ and RIC8A (G_q); $G\alpha_{12}$-Lg, Sm-$G\beta_1$, $G\gamma_2$ and RIC8A (G_{12}); $G\alpha_{13}$-Lg, Sm-$G\beta_1$, $G\gamma_2$ and RIC8A (G_{13}) After 1-day culture, the transfected cells were harvested with 1 mL of 0.53

mM EDTA-containing Dulbecco's PBS (D-PBS), followed by addition of 2 mL the HEPES-containing HBSS. The cells were centrifuged at 190 g for 5 min and resuspended in 2 mL of the 0.01% BSA- and 5 mM HEPES (pH 7.4)-containing HBSS (assay buffer). The cell suspension was seeded in a 96-well culture white plate (Greiner Bio-One) at a volume of 80 µl (per well hereafter) and loaded with 20 µl of 50 µM coelenterazine (Carbosynth) solution diluted in the assay buffer. After 2-hour incubation with coelenterazine at room temperature, background luminescent signals were measured using a luminescent microplate reader (SpectraMax L, Molecular Devices). Test compound (6X, diluted in the assay buffer) was manually added to the cells (20 µl). Luminescent signals were measured 3-5 min after ligand addition and divided by the initial count. The ligand-induced signal ratio was normalized to that treated with vehicle. Concentration-response curves were fitted to all data by the Nonlinear Regression: Variable slope (four parameter) in the Prism 9 tool with a constraint of the Hill Slope of absolute value less than 2.

Flow cytometry

Flow cytometry analysis was performed as previously described[18]. Plasmid transfection was performed in a 12-well plate with volumes of 500 ng plasmid encoding N-terminally FLAG epitope-tagged GPCR. The transfected cells were harvested by adding 300 µl of 0.53 mM EDTA-containing D-PBS, followed by 300 µl of 5 mM HEPES (pH 7.4)-containing HBSS. The cell suspension was dispensed in a 96-well V-bottom plate (200 µl per well, two wells per sample). After centrifugation at 700 g for 1 min, the cells were washed once with D-PBS and pelleted. Cell pellets were suspended in 2% goat serum- and 2mM EDTA-containing D-PBS (blocking buffer; 100 µl per well) and incubated for 30 min on ice. After centrifugation at 700 g for 1 min, the cells were stained with anti-FLAG epitope tag monoclonal antibody (Clone 1E6, FujiFilm Wako Pure Chemicals; 10 mg/ml in the blocking buffer; 50 µl per well) for 30 min on ice. After rinse with D-PBS, cells were labeled with a goat anti-mouse IgG secondary antibody conjugated with Alexa Fluor 488 (Thermo Fisher Scientific; 10 mg/ml dilution in the blocking buffer; 25 µl per well) for 15 min on ice. The cells were washed once with D-PBS, resuspended in 100 µl of 2 mM EDTA-containing-D-PBS and filtered through a 40 mm filter. The fluorescently labeled cells (approximately 20,000 cells per sample) were analyzed by an EC800 flow cytometer (Sony). Fluorescent signal derived from Alexa Fluor 488 was recorded in an FL1 channel and flow cytometry data were analyzed by a FlowJo software (FlowJo). Values of mean fluorescence intensity (MFI) were used for quantification.

Animals

All mice were kept on a 12-h light/12-h dark cycle (09:00-21:00) at controlled temperature (23 ºC) and free access to water and food. Mice were maintained on a standard mouse chow (CE-2, CLEA Japan Inc.)

or were switched to a high-fat diet (High Fat Diet 32, CLEA Japan inc., 60% kcal fat, 5.1 kcal/gram) when they were 6 weeks old. All experiments were conducted using 8-14-week-old males.

Generation of adipo-G_{12}D mice

A fragment encoding HA-tagged G_{12}D was amplified by polymerase chain reaction (Fwd: 5'-ACATTATACGAAGTTATCGGCGCGCCGCCACCATGTACCCATAC-3', Rev: 5'-CGAATTGATCGCGGCCGCGGCGCGCCTCACAAGGCCTGCTCGGG-3') and inserted into the pR26 CAG/GFP Asc vector at AscI site using NEBuilder HiFi DNA Assembly system (New England Biolabs). The resulting targeting construct was confirmed by sequence. The targeting construct, Cas9 protein (NEB, Cat No.M0646T), crRNA (5'-ACUCCAGUCUUUCUAGAAGAguuuuagagcuaugcuguuuug-3') and tracrRNA (5'-AAACAGCAUAGCAAGUUAAAAUAAGGCUAGUCCGUUAUCAACUUGAAAAAGUGGCACCGAGUCGGUGCU-3') were microinjected into the pronuclei of fertilized ova from C57BL/6N mice using standard transgenic techniques[4]. The presence of the *ROSA26-LSL-G12DREADD-IRES-GFP* transgene in the F0 mice genome was confirmed via PCR analysis of mouse tail DNA (Fwd: 5'-CTGCCCGAGCGGAAACGCCACTGAC-3', Rev: 5'-CCTGGACTACTGCGCCCTACAGA-3'). We generated adipo-G_{12}D mice by crossing *ROSA26-LSL-G12D-IRES-GFP* mice with *Adipoq-Cre* mice (The Jackson Laboratory; stock no. 028020). All experiments were carried out with male littermates. *ROSA26-LSL-G12D-IRES-GFP* mice that lacked the *Adipoq-Cre* transgene served as control animals in all experiments.

Western blotting

Adipose tissues were homogenized in RIPA buffer (50 mM Tris-HCl (pH 7.4), 150 mM NaCl, 1% Triton X-100, 0.5% sodium deoxycholate, 0.1% SDS, 1 mM EDTA, and 1 mM phenylmethylsulfonyl fluoride). Protein concentrations of the lysates were measured by the Pierce BCA Protein Assay Kit (Thermo Fisher Scientific) and adjusted by adding RIPA buffer. Lysates were denatured by SDS-PAGE sample buffer (62.5 mM Tris-HCl (pH 6.8), 50 mM dithiothreitol, 2% SDS, 10% glycerol and 4 M urea, 1 mM EDTA and 1 mM phenylmethylsulfonyl fluoride) and were separated by 12.5% SDS-polyacrylamide gel electrophoresis. Subsequently, the proteins were transferred to nitrocellulose membrane (GE Healthcare). The blotted membrane was blocked with 5% skim milk-containing blotting buffer (10 mM Tris-HCl (pH 7.4), 190 mM NaCl and 0.05% Tween 20), immunoblot with primary and secondary antibodies. Primary antibodies used in this study were anti-HA-tag rabbit monoclonal antibody (Cell Signaling Technology, #3724, lot 10, 1:1000) and anti-UCP1 rabbit polyclonal antibody (Sigma Aldrich, U6382, Batch

0000109635, 1:1000). Secondary antibodies conjugated with horseradish peroxidase (HRP) were anti-Rabbit IgG, HRP-Linked F(ab')$_2$ Fragment Donkey (GE Healthcare, NA9340, lot 17041889). Membrane was soaked with a luminol reagent (100 mM Tris-HCl (pH 8.5), 50 mg per mL Luminol Sodium Salt HG (FujiFilm Wako Pure Chemical), 0.2 mM *p*-Coumaric acid and 0.03% (v/v) of H_2O_2). A chemiluminescence image was acquired and band intensity was quantified with Amersham Imager 680 (Cytiva).

Quantitative RT-PCR analysis

Total RNA from adipose tissue was isolated using a FastGene RNA Basic kit (NIPPON Genetics) and then reverse-transcribed with a High-Capacity cDNA RT Kits (Applied Biosystems) according to the manufacturer's instructions. PCRs were performed with TB Green® Premix Ex Taq™ II (Tli RNaseH Plus) (Takara Bio) and monitored by ABI Prism 7300 (Applied Biosystems). RNA expression data were normalized relative to the expression of *B2m*. The PCR primer sequences are listed in Supplementary Table 1.

Histology

Adipose tissues were fixed in 4% paraformaldehyde for 24 h and embedded in paraffin. Five-μm-thick sections were prepared, and then stained with H&E. Bright-field images of the stained tissue sections were taken with Zeiss Axio Imager equipped with visualix V310B (visualix).

Image analysis

Sections were stained with H&E and image analysis was performed by ImageJ Plugin 'MRI_Adipocyte_Tools' (https://dev.mri.cnrs.fr/projects/imagej-macros/wiki/ Adipocytes_Tool). Adipocytes were manually circled and their numbers were measured. Over 100 cells were detected in each individual for the quantification. For lipid droplet area quantification, unstained (white color) area was identified as the lipid droplet area and measured using the ImageJ software.

***in vivo* metabolic test**

For acute CNO challenge tests, mice that had been fasted for 4 h (0900-1300) were injected i.p. with 1 mg/kg CNO dihydrochloride. Blood was collected from the tail vein before and at specific time points after CNO treatment. Plasma NEFA and glucose levels were determined using a LabAssay^{TM} NEFA (Wako) and LabAssay^{TM} Glucose (Wako), respectively. Plasma insulin levels were measured using AlphaLISA insulin immunoassay kit (PerkinElmer). Intraperitoneal glucose tolerance tests were

performed with mice that had been fasted overnight for 16 h (1800-1000). Blood glucose concentrations were measured before and after i.p. injection with glucose (1 or 2 g/kg, as indicated). For insulin tolerance tests (ITT), mice that had been fasted for 4 h (0900-1300) were injected i.p. with human insulin (0.75 U/kg, Humulin, Eli Lilly). For chronic CNO treatment experiments, mice that had been maintained on a HFD for 4 weeks were injected with CNO daily (1 mg/kg i.p.) and CL316,243 three times per week for 2 weeks. During the CNO injection period, the mice continued to consume the same HFD as before.

Induction of WAT browning

For β3AR-induced WAT browning, mice were injected i.p. with 1 mg/kg CL316,243 daily for five days and analyzed at 24 h after the last injection.

RNA sequencing

Mice were injected with CNO dihydrochloride (1 mg/kg, i.p.) and CL316,243 (1 mg/kg, i.p.) daily for 5 days. Twenty-four hours after the last injection, total RNA was extracted from iWAT using FastGene RNA Premium Kit (NIPPON Genetics) according to the manufacturer's instructions. Sequencing library preparation and RNA sequencing was carried out at Novogene Bioinformatics Technology Co., Ltd., in Beijing, China. Sequencing libraries were prepared by NEBNext Ultra RNA LP Kit (NEB) and RNA sequencing was performed on novaSeq 6000 (illumina). The RNA-seq data are viewable under DDBJ accession number PRJDB14356. Sequencing Fastq files were uploaded to and processed with RaNA-seq (https://ranaseq.eu) to obtain TPM values for each gene. Gene Set Enrichment Analysis (GSEA) was performed against Hallmark gene sets in MSigDB using GSEA 4.2.3 software (https://www.gsea-msigdb.org/gsea/index.jsp) with 1000 gene-set permutations. The gene sets were considered as significantly enriched if FDR q-value < 0.05.

Body temperature

Mice at 8–9 weeks of age were single-caged and received daily injections of CNO dihydrochloride (1 mg/kg i.p., daily) and CL316,243 (1 mg/kg i.p., daily) for 5 days. Immediately after the last injection at 10 a.m., the mice were transferred to 4 ºC. Body temperature was measured at 0, 1, 3, 9 h using a rectal thermometer (Physitemp; BAT-12).

VO_2 measurement

To analyze the contribution of $G_{12}D$ to energy expenditure, whole animal oxygen consumption was examined. Mice were injected with CNO dihydrochloride (1 mg/kg, i.p., daily) and CL316,243 (1 mg/kg,

i.p., daily) for 5 days. The mice were placed in metabolic chambers and followed 16 h later prior to another injection (CNO and CL316,243; 1 mg/kg each, i.p.) at 10 a.m. Energy expenditure was measured using indirect calorimetry (MK-5000RQ; Muromachi). The chamber volume was 720 ml, the airflow to the chamber was 700 mL/min, and samples were taken every 3 min.

Measurement of mouse plasma NEFA, glycerol, and glucose levels

Mice that had been fasted for 4 h (0900-1300) were injected i.p. with CNO dihydrochloride (1 mg/kg, i.p.) and CL316,243 (1 mg/kg, i.p.). Blood was collected from the tail vein before and at specific time points after CNO and CL316,243 treatment. Plasma NEFA and glucose levels were determined as described above. Plasma glycerol level was measured using LabAssay™ Triglyceride (Wako) in the presence of a lipase inhibitor orlistat at 10 μM (Supplementary Figure 6a).

Primary adipocyte culture and differentiation.

Isolation of SVF and induction for beige adipocytes were performed as previously described with minor modifications[5]. Briefly, iWAT tissues were subjected to enzymatic digestion with 1.5 mg/ml collagenase D (Roche) and 2.4 U/mL dispase II (Roche) at 37°C for 50 min. After digestion, the cell suspension was centrifuged and the resulting pellet, called SVF, was resuspended in the complete medium (Dulbecco's modified Eagle's medium/F12 (Sigma Aldrich) containing 1.2g/L $NaHCO_3$ and 10% FCS). The cells were then filtered through a 70 μm nylon cell strainer (Greiner). To remove contaminated cells, SVFs were plated in a collagen-coated dish and 3-h later, the cells were rinsed twice with PBS. For beige adipocyte induction, SVFs were seeded in a 12-well plate (8×10^4 cells/well) and cultured to confluence for 2 days. Differentiation was induced by a complete medium containing 0.125 mM indomethacin, 5 μM dexamethasone, 0.5 mM 3-isobutyl-1-methylxanthine, 0.5 μM rosiglitazone, 5 μg/mL insulin, and 1 nM T3 for 48 h, followed by treatment with insulin, T3, and rosiglitazone for another 6 days, with medium replacement every 2 days. CNO was added to the culture medium and the cells were cultured for another 1 day. Inhibitors were added to the well 30 minutes before CNO stimulation.

GPCR expression study

The RNA-seq data (GSE131861) were uploaded to and analyzed by the RaNA-seq (https://ranaseq.eu). TPM values are given as mean value (n = 6). G_{12}-coupled GPCRs were identified based on IUPHAR/BPS Guide to Pharmacology Database and two published literatures[2,6].

Statistics

Data were analyzed by using the Prism 9 software (GraphPad) and Microsoft Excel (Microsoft) software. All data are expressed as means ± s.e.m. for the indicated number of independent experiments or observations. Data were then tested for statistical significance by the one-way or the two-way ANOVA, followed by the indicated post-hoc tests, or by the two-tailed unpaired Student's *t*-test, as appropriate. *P*-values less than 0.05 were considered as statistically significant. The statistical tests that were used are indicated in the figure legends.

Supplementary References

1. Chu, V. T. *et al.* Efficient generation of Rosa26 knock-in mice using CRISPR/Cas9 in C57BL/6 zygotes. *BMC Biotechnol.* **16**, 1–15 (2016).
2. Inoue, A. *et al.* Illuminating G-Protein-Coupling Selectivity of GPCRs. *Cell* **177**, 1933-1947.e25 (2019).
3. Körber, J., Löffler, S., Schollmeyer, D. & Nubbemeyer, U. Synthesis and oxidant properties of phase 1 benzepine N-oxides of common antipsychotic drugs. *Synth.* **45**, 2875–2887 (2013).
4. Aida, T. *et al.* Cloning-free CRISPR/Cas system facilitates functional cassette knock-in in mice. *Genome Biol.* **16**, (2015).
5. Abe, Y. *et al.* Histone demethylase JMJD1A coordinates acute and chronic adaptation to cold stress via thermogenic phospho-switch. *Nat. Commun.* **9**, (2018).
6. Avet, C. *et al.* Effector membrane translocation biosensors reveal G protein and Parrestin coupling profiles of 100 therapeutically relevant GPCRs. *Elife* **11**, 1–34 (2022).

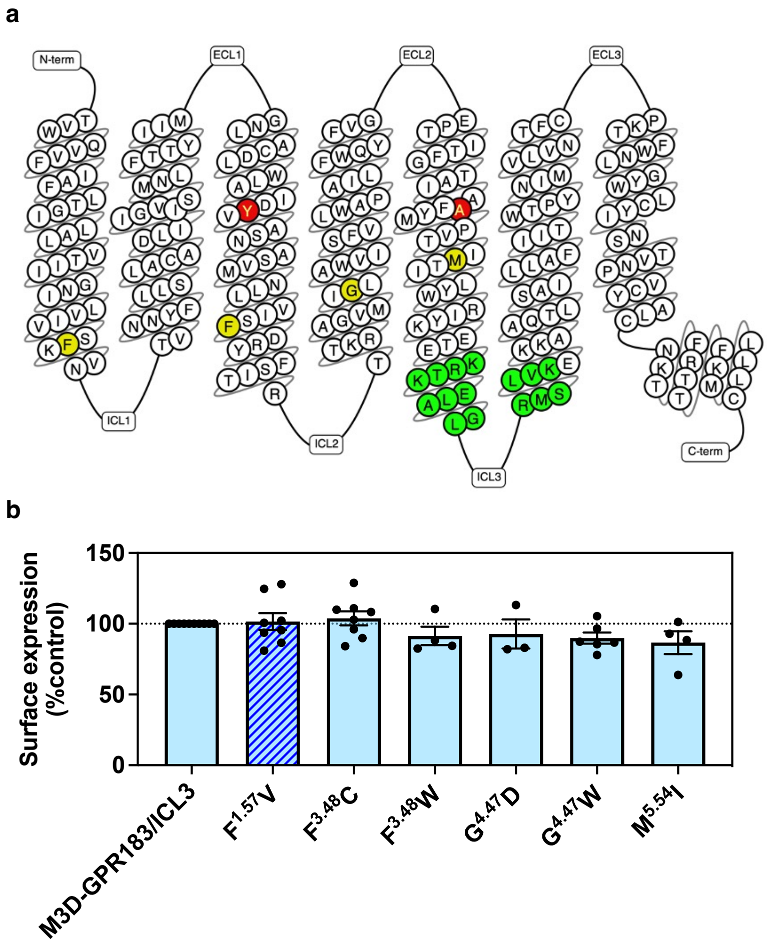

Supplementary Figure 1. Surface expression of the M3D-GPR183/ICL3-derived constructs

a, Snake plot representation of the human M3 receptor obtained from the GPCRDB (https://www.gpcrdb.org). The residues commonly mutated in the M3 receptor-based DREADD ($Y^{3.33}C$ and $A^{5.46}G$) are highlighted in red and the residues selected by PRECOG ($F^{1.57}$, $F^{3.48}$, $G^{4.47}$, $M^{5.54}$) are highlighted in yellow. Note that intracellular loop 3 (ICL3) and a part of TM5 and TM6 of hM3D highlighted in green are substituted by ICL3 of GPR183 (amino-acid sequence: RTAKQNPLTEKSGV) in M3D-GPR183/ICL3 construct.

b, Surface expression analysis for the M3D-GPR183/ICL3 mutants. HEK293 cells transiently expressing the indicated M3D-GPR183/ICL3 construct were subjected to the flow cytometry analysis using an anti-FLAG epitope-antibody, followed by a fluorescently labeled secondary antibody (n = 3-10 per group). Bars and error bars represent mean and SEM, respectively. Statistical significance was determined by one-way ANOVA followed by the Dunnett's post-hoc test. No line or asterisk is marked if the P-value is greater than 0.05.

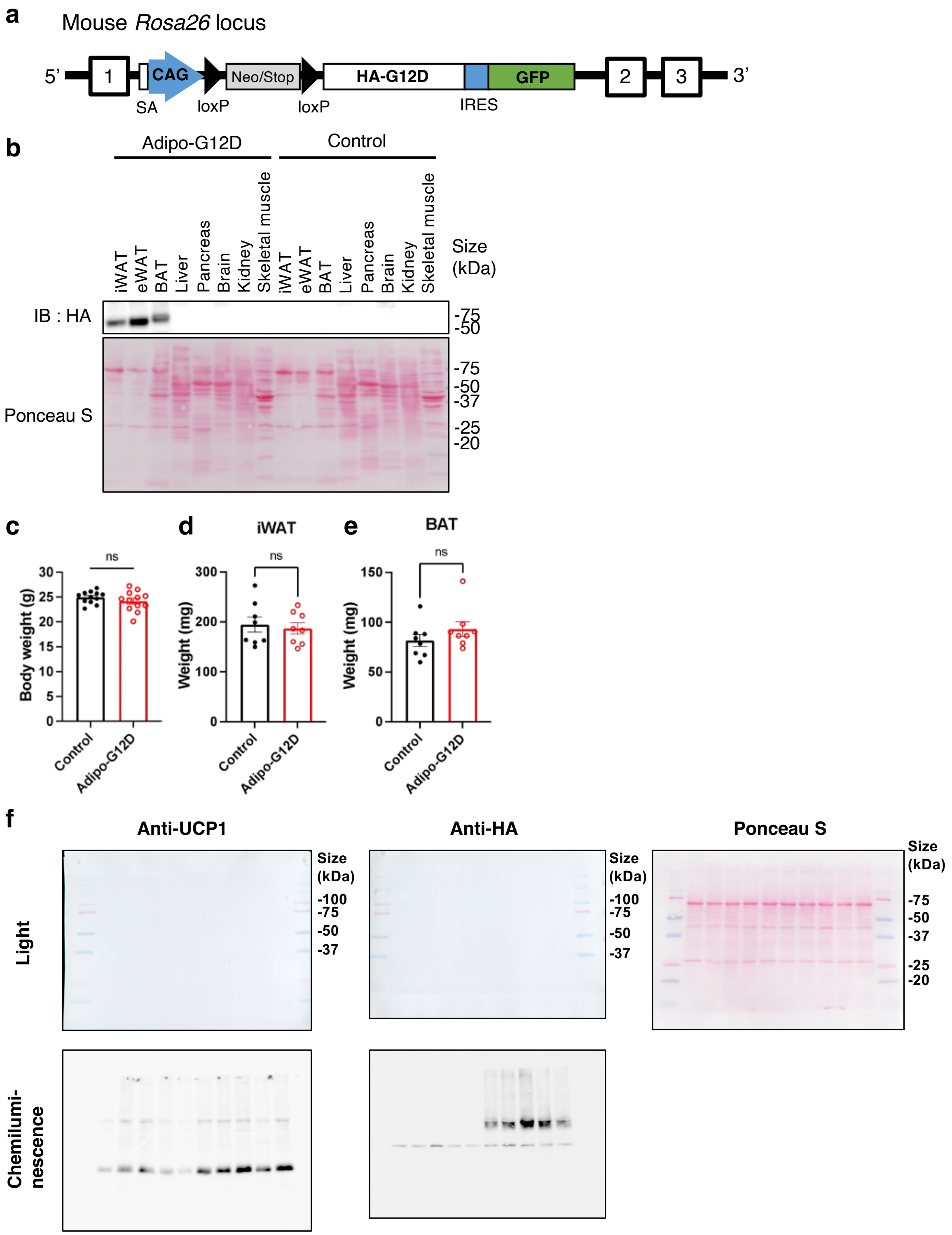
a
Mouse Rosa26 locus
5'
1
CAG
Neo/Stop
HA-G12D
GFP
2
3
3'
SA
loxP
loxP
IRES
b
Adipo-G12D
Control
iWAT
eWAT
BAT
Liver
Pancreas
Brain
Kidney
Skeletal muscle
Size (kDa)
IB : HA
Ponceau S
-75
-50
-37
-25
-20
c
Body weight (g)
ns
Control
Adipo-G12D
d
iWAT
Weight (mg)
ns
e
BAT
f
Anti-UCP1
Anti-HA
Ponceau S
Light
Chemilumi-nescence
-100
-75
-50
-37
-25
-20

Supplementary Figure 2. Validation of the adipo-$G_{12}D$ mice

a, Schematic representation of the *ROSA26-LSL-G12D-IRES-GFP* allele. SA: splicing acceptor; CAG: CAG promoter; Neo/Stop: Neomycin resistant gene and a stop codon cassette; HA-$G_{12}D$: HA-epitope tag-fused $G_{12}D$; IRES: internal ribosome entry site; GFP: green fluorescent protein.

b, Western blot analysis and Ponceau S staining showing adipose tissue-specific $G_{12}D$ expression in the adipo-$G_{12}D$ mice.

c-e, Body weight (b), iWAT weight (c), and BAT weight (d) of the control and the adipo-$G_{12}D$ mice maintained on RC for eight weeks (n = 8 per group). Statistical significance was determined by the two-tailed Student's t test.

f, Uncropped images of Figure 1i.

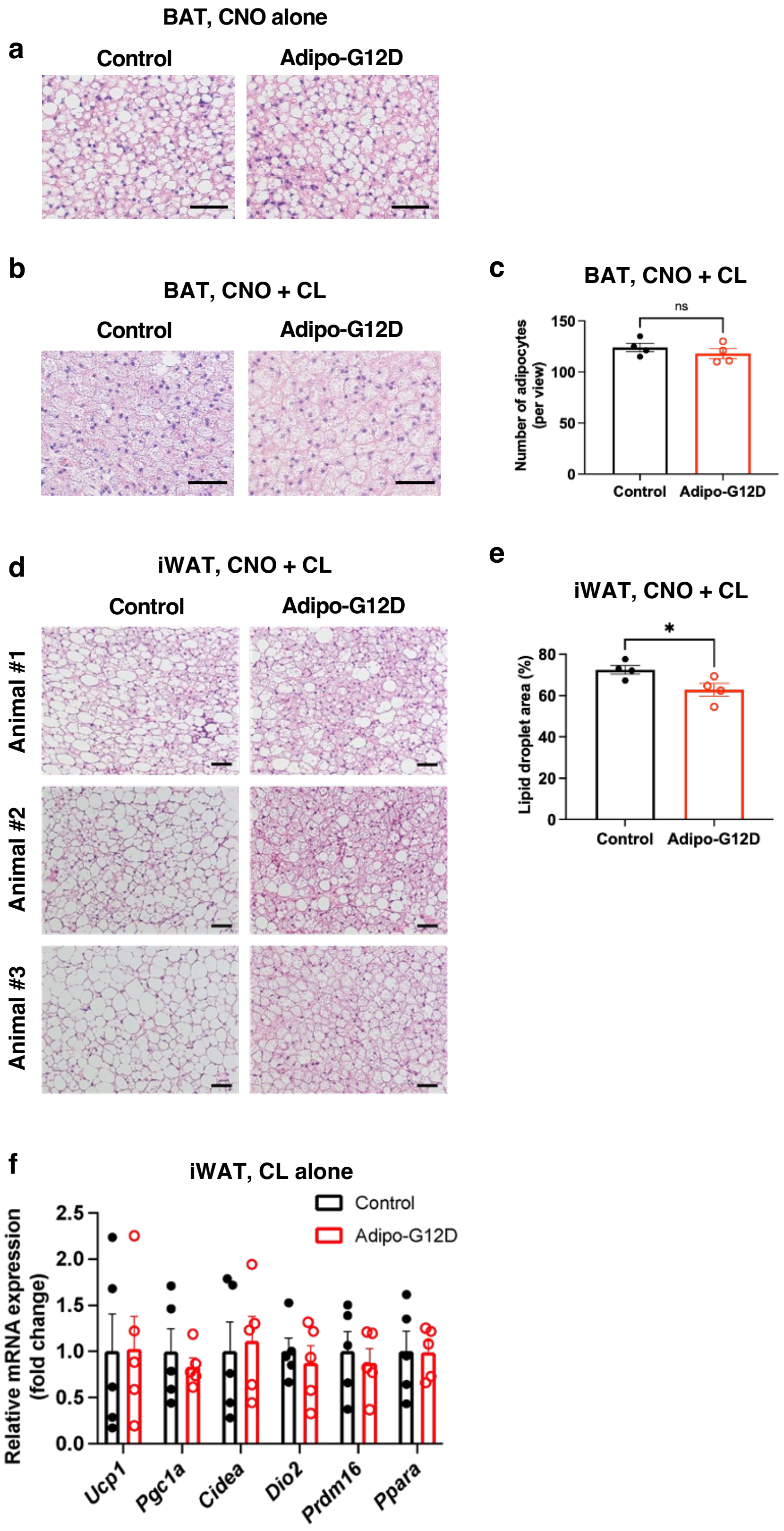
a
BAT, CNO alone
Control
Adipo-G12D
b
BAT, CNO + CL
Control
Adipo-G12D
c
BAT, CNO + CL
ns
Number of adipocytes (per view)
150
100
50
0
Control
Adipo-G12D
d
iWAT, CNO + CL
Control
Adipo-G12D
Animal #1
Animal #2
Animal #3
e
iWAT, CNO + CL
*
Lipid droplet area (%)
80
60
40
20
0
Control
Adipo-G12D
f
iWAT, CL alone
Control
Adipo-G12D
Relative mRNA expression (fold change)
2.5
2.0
1.5
1.0
0.5
0.0
Ucp1
Pgc1a
Cidea
Dio2
Prdm16
Ppara

Supplementary Figure 3. Histological evaluation of adipocyte morphology in BAT

a, b, Representative H&E staining of BAT following CNO alone (1 mg/kg, i.p., daily) (a) or CNO with CL316,243 administration (1 mg/kg each, i.p., daily) for 5 days (b) (scale bar: 50 μm).

c, Quantification of adipocyte numbers in the BAT sections. *n* = 4 per group.

d, Representative H&E staining of iWAT from three animals for each genotype following CNO with CL316,243 administration (1 mg/kg each, i.p., daily) for 5 days (scale bar: 50 μm).

e, Quantification of lipid droplet ares of the iWAT sections. *n* = 4 per group.

f, Expression of thermogenic genes in iWAT following CL administration alone (1 mg/kg, i.p., daily) for five days (*n* = 5 per group).

In the figure panels (c, e, f), bars and error bars represent mean and SEM, respectively. Statistical significance was determined by the two-tailed Student's *t*-test (c, e) and the two-way ANOVA (f). ns, $P > 0.05$ (c); *, $P < 0.05$ (e). f, there was no significant difference between the groups.

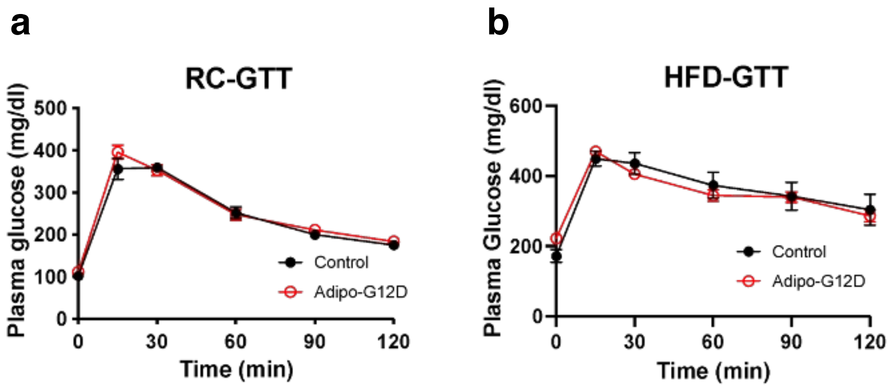

Supplementary Figure 4. Acute $G_{12}D$ activation did not affect glucose metabolism.

a, Intraperitoneal glucose tolerance test of the control and the adipo-$G_{12}D$ mice maintained on RC. CNO (1 mg/kg, i.p.) was injected with glucose (2 g glucose/kg i.p.) simultaneously (n = 6 for the control and n = 5 for the adipo-$G_{12}D$).

b, Intraperitoneal glucose tolerance test of the control and the adipo-$G_{12}D$ mice maintained HFD for 6 weeks. CNO (1 mg/kg, i.p.) was injected with glucose (1 g glucose/kg, i.p.) simultaneously (n = 7 for the control and n = 5 for the adipo-$G_{12}D$).

In all figure panels, symbols and error bars represent mean and SEM, respectively. Statistical significance was determined by the two-way ANOVA followed by the Sidak's post-hoc test. No line or asterisk is marked if the *P*-value is greater than 0.05.

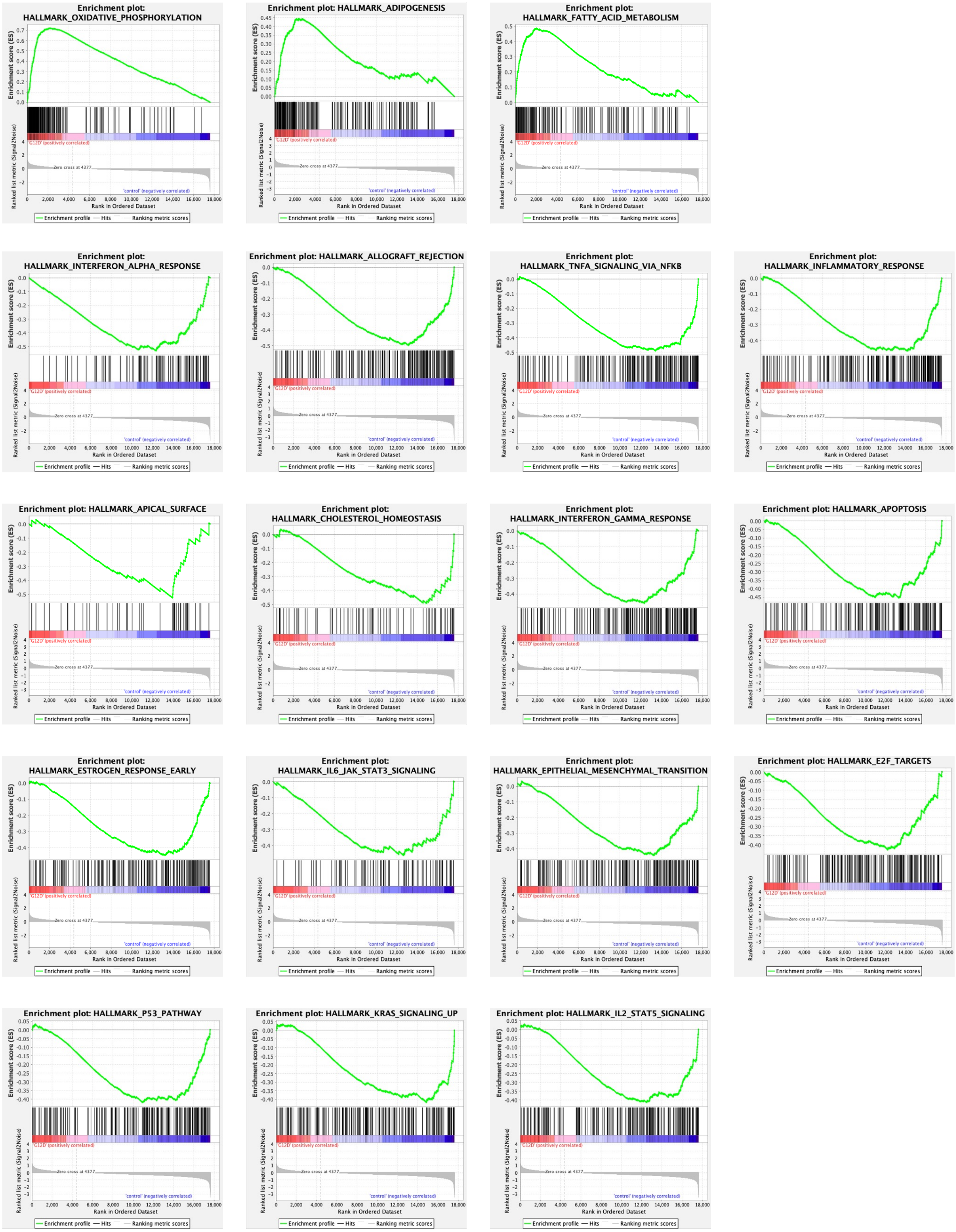

Enrichment plot: HALLMARK_OXIDATIVE_PHOSPHORYLATION
Enrichment plot: HALLMARK_ADIPOGENESIS
Enrichment plot: HALLMARK_FATTY_ACID_METABOLISM
Enrichment plot: HALLMARK_INTERFERON_ALPHA_RESPONSE
Enrichment plot: HALLMARK_ALLOGRAFT_REJECTION
Enrichment plot: HALLMARK_TNFA_SIGNALING_VIA_NFKB
Enrichment plot: HALLMARK_INFLAMMATORY_RESPONSE
Enrichment plot: HALLMARK_APICAL_SURFACE
Enrichment plot: HALLMARK_CHOLESTEROL_HOMEOSTASIS
Enrichment plot: HALLMARK_INTERFERON_GAMMA_RESPONSE
Enrichment plot: HALLMARK_APOPTOSIS
Enrichment plot: HALLMARK_ESTROGEN_RESPONSE_EARLY
Enrichment plot: HALLMARK_IL6_JAK_STAT3_SIGNALING
Enrichment plot: HALLMARK_EPITHELIAL_MESENCHYMAL_TRANSITION
Enrichment plot: HALLMARK_E2F_TARGETS
Enrichment plot: HALLMARK_P53_PATHWAY
Enrichment plot: HALLMARK_KRAS_SIGNALING_UP
Enrichment plot: HALLMARK_IL2_STAT5_SIGNALING
Enrichment score (ES)
Ranked list metric (Signal2Noise)
'G12D' (positively correlated)
Zero cross at 4377
'control' (negatively correlated)
Rank in Ordered Dataset
Enrichment profile
Hits
Ranking metric scores

Supplementary Figure 5. GSEA analysis of iWAT from the control and the adipo-$G_{12}D$ mice treated with CNO and CL316,243 daily for five days.

Graphs showing the result of GSEA analysis of significantly enriched gene sets. The figure panels were generated through the online GSEA 4.2.3 software (https://www.gsea-msigdb.org/gsea/index.jsp).

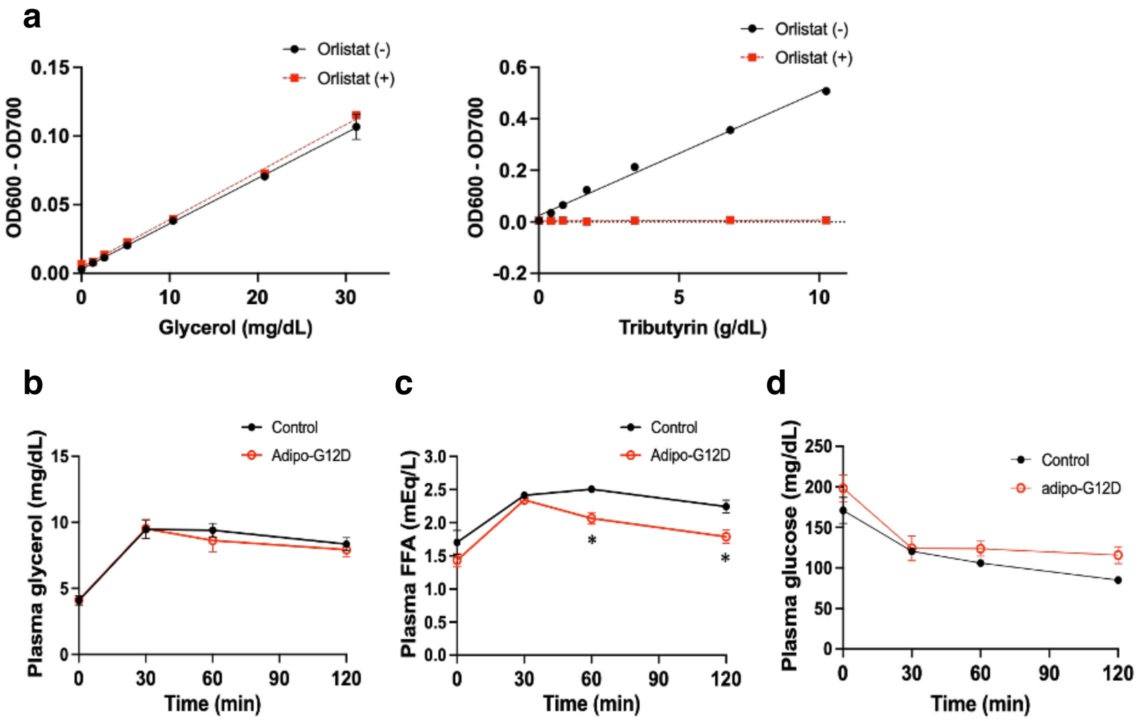

Supplementary Figure 6. Plasma glycerol, free-fatty-acid and glucose levels upon CNO and CL316,243 administration.

a, Validation of the glycerol measurement. Represntative standard curves (4 wells per concentration) of glycerol and tributyrin using the LabAssay^{TM} Triglyceride kit in the presence or absence of the lipase inhibitor orlistat.

b-d, Plasma was collected from the control and the adipo-G_{12}D mice before (0 min) and after (30, 60 and 120 min) CNO and CL316,243 single administration (1 mg/kg each). Glycerol (b), free-fatty-acid (c) and glucose (d) levels were measured (n = 5 per group).

In all figure panels, symbols and error bars represent mean and SEM, respectively. In the figure panes (b-d), statistical significance was determined by the two-way ANOVA followed by the Sidak's post-hoc test. No line or asterisk is marked if the P-value is greater than 0.05. $^{*}P < 0.05$

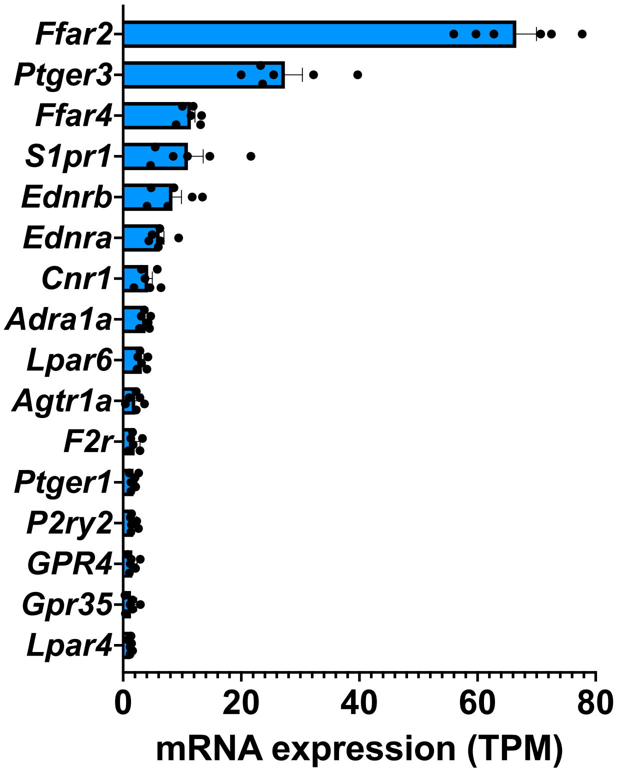

Supplementary Figure 7. G12-coupled GPCRs expressed in human and murine iWAT

TPM values for the genes encoding G12-coupled GPCRs in murine iWAT adipocytes were shown. Bars and error bars represent mean and SEM (n = 6), respectively.

Supplementary Table 1. RT-PCR primers used in this study.

Gene	Forward primer (5'→3')	Reverse primer (5'→3')
G12D	GTACCACCGATGACCCTCTG	CCAGGATGTTGCCGATGATG
Adrb3	ACCGCTCAACAGGTTTGATG	ATCCATAGCCGTTGCTTGTC
B2m	GTGACCCTGGTCTTTCTGGT	GTATGTTCGGCTTCCCATTC
Ucp1	CTTTGCCTCACTCAGGATTGG	ACTGCCACACCTCCAGTCATT
Pgc1a	AGCCGTGACCACTGACAACGAG	GCTGCATGGTTCTGAGTGCTAAG
Cidea	CACGCATTTCATGATCTTGGA	GTTGCTTGCAGACTGGGACAT
Prdm16	CAGCACGGTGAAGCCATTC	GCGTGCATCCGCTTGTG
Dio2	GGAACAGCTTCCTCCTAGATGC	TTCTCCGAGGCATAATTGTTACCTG
Pparg	GTACTGTCGGTTTCAGAAGTGCC	ATCTCCGCCAACAGCTTCTCCT
Adipoq	TTCTCCTCATTTCTGTCTGTACG	ACGTCATCTTCGGCATGACTG
Fabp4	ACACCGAGATTTCCTTCAAACTG	CCATCTAGGGTTATGATGCTCTTCA
Elovl3	GGTTGTTGAACTGGGAGACACG	CACCCGAAGGCACTTTGTTC
Ppara	TGGGCAAGAGAATCCACGAAG	TGTATGACAAAAGGCGGGTTG
